# Antimicrobial-laden PerOssal beads display in vitro inhibition and killing of extensively drug-resistant Gram-negative bacteria associated with conflict wounds in Ukraine

**DOI:** 10.1302/2046-3758.158.BJR-2026-0211.R1

**Published:** 2026-08-20

**Authors:** Henry Adam Claireaux, Edward J. A. Douglas, Scott J. C. Pallett, Luke S. P. Moore, Matthew K. O'Shea, Olena V. Moshynets, Julian R. Jones, Arul Ramasamy, Andrew M. Edwards

**Affiliations:** 1 Department of Materials, Imperial College London, South Kensington Campus, London, UK; 2 Centre for Bacterial Resistance Biology, Imperial College London, South Kensington Campus, London, UK; 3 Department of Infectious Disease, Imperial College London, South Kensington Campus, London, UK; 4 Academic Department of Military Trauma & Orthopaedics, Research & Clinical Innovation, ICT Centre, Birmingham, UK; 5 Clinical Infection Department, Chelsea and Westminster Hospital NHS Foundation Trust, London, UK; 6 Centre of Defence Pathology, Royal Centre for Defence Medicine, Queen Elizabeth Hospital, Birmingham, UK; 7 National Institute for Health Research Imperial Biomedical Research Centre, Imperial College London, Hammersmith Campus, London, UK; 8 Department of Immunology and Immunotherapy, College of Medicine and Health, University of Birmingham, Birmingham, UK; 9 Academic Department of Military Medicine, Research & Clinical Innovation, ICT Centre, Birmingham, UK; 10 Institute of Molecular Biology and Genetics of National Academy of Sciences of Ukraine, Kyiv, Ukraine; 11 Centre for Injury Studies, Imperial College, White City Campus, London, UK

**Keywords:** Open fracture, Wound, Infection, Resistance, Antimicrobial, Gram-negative bacteria, wounding, infections, Gentamicin, strains, hydroxyapatite, open fractures, calcium sulphate, calcium sulphate, bacteria

## Abstract

**Aims:**

Open fractures sustained in contemporary conflict are associated with high-energy transfer, contamination with resistant bacteria, and delays to surgical care, resulting in high rates of infection. Similar conditions occur in civilian disasters, where crush injuries and overwhelmed healthcare systems prolong the interval to surgical wound excision. Prophylactic systemic antimicrobial regimens may be ineffective due to antimicrobial resistance or limited bioavailability in bone and joint tissues. Local antimicrobial delivery can provide higher drug concentrations within the wound, which may be sufficient to overcome resistance. This route is established in bone and joint infection, although evidence in conflict trauma is lacking. We evaluated the in vitro activity of antimicrobial-laden PerOssal hydroxyapatite and calcium sulphate beads.

**Methods:**

Bacterial isolates from Ukraine isolated from contaminated traumatic wounds were tested, including extensively drug-resistant (XDR) *Klebsiella pneumoniae, Acinetobacter baumannii, Pseudomonas aeruginosa*, and *Escherichia coli*. The antimicrobial effects of antimicrobial-laden PerOssal beads were assessed by zone-of-inhibition testing and time-kill analysis.

**Results:**

All Gram-negative isolates from Ukraine were resistant to gentamicin, tobramycin, co-amoxiclav, cefazolin, and ciprofloxacin, with minimum inhibitory concentrations (MICs) exceeding European Committee on Animicrobial Susceptibility Testing (EUCAST) breakpoints. PerOssal beads laden with gentamicin, tobramycin, or cefazolin were weakly growth inhibitory. However, gentamicin–rifampicin in combination achieved consistent broad-spectrum bacterial inhibition and killing, while co-amoxiclav-loaded beads inhibited the growth of all bacterial strains.

**Conclusion:**

Antimicrobial-laden PerOssal hydroxyapatite and calcium sulphate beads displayed in vitro inhibition and killing of XDR Gram-negative bacteria associated with contaminated traumatic wounds. Early local antimicrobial delivery may reduce infection risk following heavily contaminated open fractures, particularly when surgical wound excision is delayed. This may be relevant to both modern conflict and civilian disaster settings. Further work is required to determine the effectiveness of this approach in vivo.

Cite this article: *Bone Joint Res* 2026;15(8):1055–1066.

## Article focus

- Heavily contaminated open fractures in austere environments, including modern conflict and civilian disasters, are frequently complicated by infection because surgical wound excision is delayed.
- Prophylactic systemic antimicrobial regimens may be ineffective due to poor tissue perfusion and antimicrobial resistance.
- We evaluated the in vitro activity of antimicrobial-laden PerOssal hydroxyapatite and calcium sulphate beads against bacteria relevant to contaminated traumatic wounds, including XDR Gram-negative organisms from Ukraine.

## Key messages

- Antimicrobial-laden PerOssal hydroxyapatite and calcium sulphate beads display in vitro killing of XDR Gram-negative bacteria associated with contaminated traumatic wounds.
- Early antimicrobial delivery may reduce infection risk when surgical wound excision is delayed.
- This approach may be relevant to both conflict and disaster medicine, where delays to surgical care are common.
- There is an urgent need for local antimicrobial delivery for the prevention and management of Gram-negative bone infection.

## Strengths and limitations

- Collaboration between Ukraine and the UK enabled investigation of clinically relevant multidrug-resistant isolates associated with contaminated traumatic wounds.
- Civilian–military and interdisciplinary collaboration between microbiologists, materials scientists, and clinicians brings together knowledge and skills to address the challenges of fracture-related infection.
- In vitro study of these infections has inherent limitations. In vivo, antimicrobial effects may be influenced by factors such as bioavailability, tissue perfusion, pH buffers, and the immune system.

## Introduction

Open fractures sustained in contemporary armed conflict are characterized by high-energy transfer and heavy environmental contamination, resulting in extensive devitalized tissue that provides a nidus for infection.^1^ Current theatres of war involve frequent use of high-energy munitions delivered by unmanned aerial systems and artillery.^2^ Injured servicepersons would ideally receive prophylactic systemic antimicrobials soon after the point of wounding, before being evacuated to surgical facilities for life and limb saving treatment, including wound excision.^3^ However, in large-scale combat operations, casualty numbers may overwhelm medical supply chains and available surgical capacity; treatment facilities cannot be positioned too far forward due to force protection considerations and the limited availability of trained surgical teams. Combined with denied or contested air evacuation, these factors frequently delay transfer to surgery.^4^ This may extend the interval between injury and surgical management from hours to days with consequent increased risk of musculoskeletal infection, amputation, and death. Open fractures sustained in natural disasters represent a similar challenge due to delayed surgical control of contaminated and devitalized tissue. During earthquakes in Turkey, patients experienced high-energy crush injuries from building collapse, prolonged entrapment, and delayed treatment in an overwhelmed healthcare system.^5-7^

High infection rates, including with extensively drug-resistant (XDR) bacteria, have been reported in the Russo-Ukrainian war and recent Middle Eastern conflicts.^8-14^ The organisms most frequently isolated from infected conflict wounds include: *Staphylococcus aureus*, *Klebsiella pneumoniae*, *Acinetobacter baumannii*, *Pseudomonas aeruginosa*, and *Escherichia coli*.^9,10,15,16^ These are of particular concern in the context of antimicrobial resistance (AMR), are represented within the ESKAPE pathogen group, and are ranked as critically important threats on the WHO bacterial pathogens priority list of drug-resistant organisms.^17,18^ Ukrainian antimicrobial guidelines for trauma prophylaxis recommend cefazolin or, in cases of delayed evacuation, oral fluoroquinolones (moxifloxacin or levofloxacin) or intravenous ertapenem.^19^ UK military guidelines recommend co-amoxiclav.^20^ Many bacterial strains isolated from recent conflicts are multidrug resistant (MDR) or XDR, particularly Gram-negative species which have high rates of resistance to these recommended agents, raising concern about the effectiveness of existing prophylactic regimens.^8,21,22^ Although resistant organisms are typically acquired by nosocomial transmission during hospital care, emerging data suggest that MDR/XDR wound infections may occur early in some circumstances, including in the first seven days following injury and prior to admission to major healthcare facilities.^10,21^ This may indicate primary environmental or endogenous contamination, for example spilled bowel contents. The continued spread of AMR is likely to create substantial challenges for deployed military medical services and civilian health systems as injured servicepersons are repatriated. Currently, British servicepersons have low levels of drug-resistant Enterobacterale carriage on pre-deployment testing.^23^

In addition to systemic prophylaxis, antimicrobials can also be delivered locally as powders, or with carriers such as calcium sulphate or polymethylmethacrylate (PMMA) cement. Local delivery permits substantially higher antimicrobial concentrations at the wound site compared with systemic administration, and does so without the adverse effects associated with systemic (intravenous or oral) dosing.^24^ In vitro studies have shown that gentamicin-eluting calcium sulphate can inhibit the growth of resistant bacterial strains, by eliciting a very high local drug concentration.^25,26^ Local delivery also avoids challenges related to bone penetration and limited vascular supply because the antimicrobial is directly in contact with the target. Local antimicrobial delivery is well established in orthopaedic surgery and is routinely used in the management of periprosthetic joint infection (PJI) and fracture-related infection (FRI).^27,28^ Currently, UK military guidelines do not advise local antimicrobial administration for prophylaxis after trauma. No randomized controlled trials (RCTs) or observational studies have specifically evaluated local antimicrobial use for wounds sustained in conflict or humanitarian settings, though related research has been conducted in sterile operating theatre environments in predominantly high-income countries.^29^ Overall, existing studies, including systematic reviews, have reported lower infection rates when local antimicrobials were used in addition to systemic antimicrobials compared with systemic therapy alone. Four RCTs have evaluated the use of local antimicrobials in the management of open limb fractures, with prevention of deep wound infection as the primary outcome, which was measured at varying timepoints.^30-33^ These RCTs have investigated vancomycin powder and PMMA beads containing vancomycin and tobramycin. However, vancomycin demonstrates no activity against Gram-negative organisms, which are a major concern in conflict-related wound infections, including in Ukraine. Studies have not identified an association between combined local and systemic antimicrobial delivery and adverse events such as nephrotoxicity or impaired bone healing.^34-40^ There is no strong evidence from clinical studies that local antimicrobial administration to open fractures contributes to antimicrobial resistance.^29^ Previous research has focused primarily on Gram-positive infections, which limits the generalizability of conclusions to MDR/XDR Gram-negative organisms associated with open fractures sustained in conflict.

**Fig. 1:**
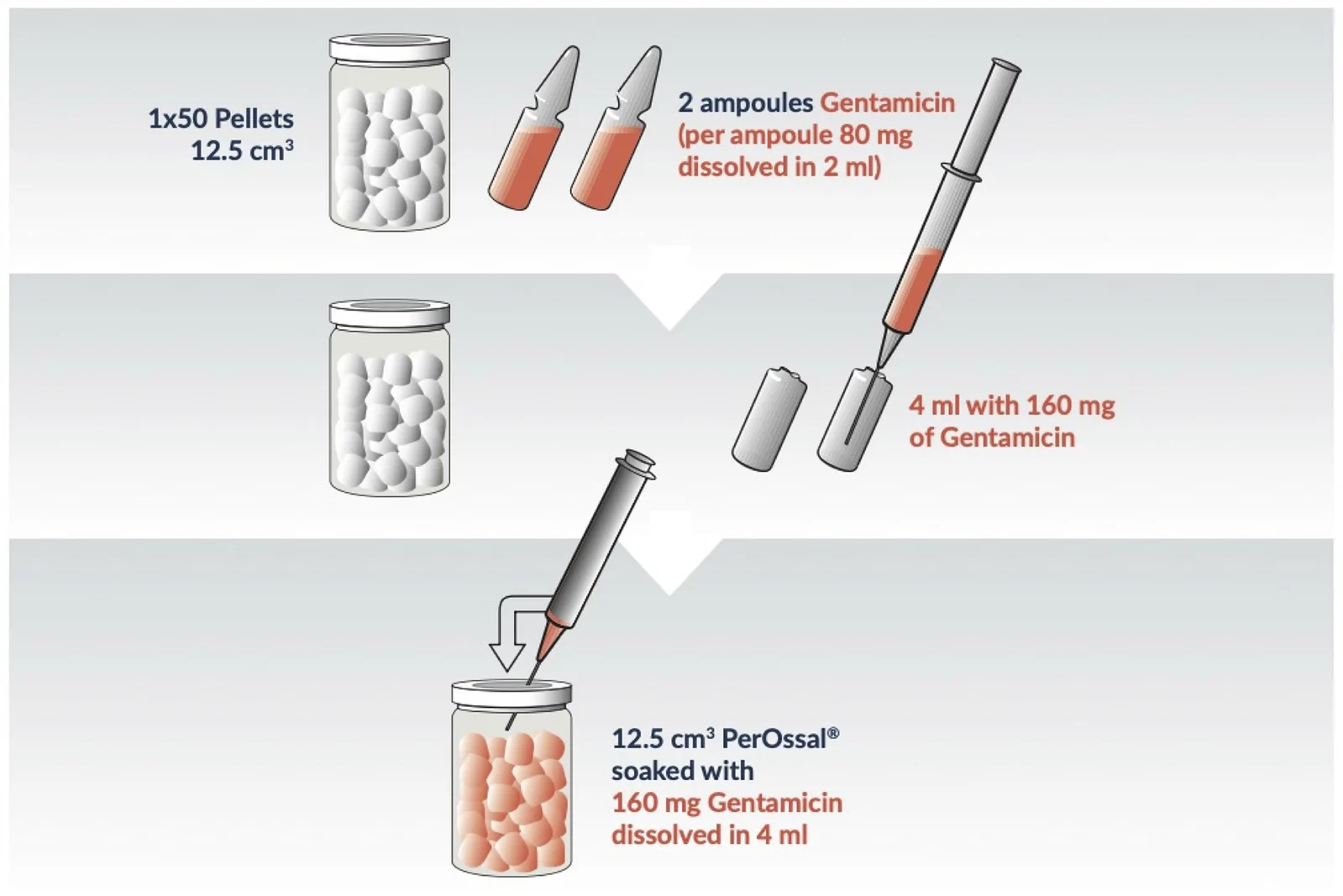
Diagram showing how PerOssal (OSARTIS, Germany) beads are made up with vials of antimicrobial for administration. Gentamicin used as an example. Reproduced with permission from OSARTIS.

Many bacteria isolated from the Russo-Ukrainian war are resistant to systemically safe and achievable antimicrobial concentrations (antibiotic breakpoint).^10,21^ However, local delivery at several orders of magnitude above the breakpoint may produce inhibitory or bactericidal effects. This is established practice in the management of FRIs and PJIs.^24^ Early administration of high-dose local antimicrobials is essential to prevent progression from initial contamination to established infection with mature biofilm formation. Musculoskeletal infections with biofilm are substantially more difficult to treat as bacterial MICs increase by ten- to 1,000-fold compared with planktonic organisms.^41^ Consequently, large surgical excisions or amputations are often required for source control. Therefore, prevention of the early establishment of these infections is of great importance. In conflict settings, local antimicrobials could be applied by a Combat Medic or Regimental Medical Officer (General Practitioner) close to the point of wounding at the time of dressing application. PerOssal (Osartis, Germany) is an antimicrobial carrier composed of porous hydroxyapatite and calcium sulphate beads (Figure 1). Predictable volumes of antimicrobial solution are absorbed over two minutes and 95% is eluted over ten days, with greater release above breakpoint MICs until day four.^42^ Resolution of infection and bone healing have been reported in clinical studies in osteomyelitis and infected nonunion fractures.^43,44^ Alternative options include drug powders, Stimulan (calcium sulphate beads; Biocomposites, UK), Cerament (calcium sulphate + hydroxyapatite setting paste; BONESUPPORT, USA) and PMMA cement. The optimal antimicrobial carrier for military use would be pre-made, heat-stable for simple logistics, compatible with a wide range of antimicrobials, and easy to apply.

We hypothesize that, when delivered early after injury and in subsequent surgical treatment, local antimicrobial delivery may reduce the impact of XDR Gram-negative infections in wounds sustained during conflict or natural disasters. This may reduce morbidity, amputations, and mortality associated with these infections.^29^ This study investigated the in vitro effects of antimicrobial-laden PerOssal against several bacterial species relevant to contaminated traumatic wounds, focusing on XDR Gram-negative organisms isolated from conflict wounds in Ukraine.

## Methods

The 15 bacterial isolates included in this study are described in Table I. Bacteria were isolated from conflict-associated wound infections in the Kharkiv, Kryvyi Rih, and Kyiv regions of Ukraine (February to May 2024). They include XDR *A. baumannii*, *K. pneumoniae*, *P. aeruginosa*, and *E. coli*.^10,22^ Additional clinical strains were selected (methicillin-resistant *S. aureus* JE2, *E. coli* CFT 073, and *E. faecalis* O1GX) to include representative Gram-positive organisms and a comparator drug susceptible *E. coli* strain.

**Table I.** Description of bacterial strains and associated minimum inhibitory concentration (minimum bactericidal concentration) (mg L^-1^). Breakpoints as per European Committee on Antimicrobial Susceptibility Testing clinical breakpoint tables.^45^

Species	Strain	Sample type	Gentamicin	Tobramycin	Co-amoxiclav	Cefazolin	Ciprofloxacin
MRSA	JE2^46^	Skin abscess	S ≤ 2 2 (2)	S ≤ 2 2 (2)	No BP 1 (4)	No BP 1 (1)	*R* > 2 4 (16)
*E. coli*	CFT 073^47^	Urinary tract	S ≤ 2 0.5 (0.5)	S ≤ 2 0.5 (0.5)	S ≤ 8 2 (4)	0.001 < I < 4 2 (4)	S ≤ 0.25 0.25 (0.5)
*E. faecalis*	O1GX^48^	Gastrointestinal tract	No BP* 4 (16)	No BP* 8 (32)	No BP 0.5 (1)	No BP 2 (4)	S ≤ 4 0.125 (> 0.5)
*K. pneumoniae*	21	Deep wound	*R* > 2 Growth at 64	*R* > 2 Growth at 64	*R* > 8 Growth at 128	*R* > 4 Growth at 64	*R* > 0.5 Growth at 4
*K. pneumoniae*	30	Wound	*R* > 2 Growth at 512	*R* > 2 Growth at 512	*R* > 8 Growth at 512	*R* ≥ 8 Growth at 64	*R* > 0.5 Growth at 16
*K. pneumoniae*	93	Deep wound	*R* > 2 Growth at 512	*R* > 2 Growth at 512	*R* > 8 Growth at 512	*R* > 8 Growth at 64	*R* > 0.5 16 (16)
*A. baumannii*	9	Wound	*R* > 4 Growth at 64	*R* > 4 Growth at 64	No BP Growth at 512	No BP Growth at 64	*R* > 1 Growth at 16
*A. baumannii*	13	Deep wound	*R* > 4 Growth at 512	*R* > 4 Growth at 512	No BP Growth at 512	No BP Growth at 64	*R* > 1 Growth at 16
*A. baumannii*	28	Wound	S ≤ 4 2 (2)	S ≤ 4 1 (1)	No BP Growth at 512	No BP Growth at 64	*R* > 1 Growth at 16
*P. aeruginosa*	36	Deep wound	No BP Growth at 512	*R* > 2 Growth at 64	No BP Growth at 512	No BP Growth at 64	*R* > 0.5 Growth at 8
*P. aeruginosa*	74	Deep wound	No BP Growth at 512	*R* > 2 Growth at 512	No BP 512 (> 512)	No BP Growth at 64	*R* > 0.5 Growth at 16
*P. aeruginosa*	102	Deep wound	No BP Growth at 512	*R* > 2 Growth at 512	No BP Growth at 512	No BP Growth at 64	*R* > 0.5 Growth at 16
*E. coli*	22	Deep wound	*R* > 2 Growth at 64	*R* > 2 64 ( > 64)	*R* > 8 128 (128)	*R* > 8 Growth at 64	*R* > 0.5 Growth at 4
*E. coli*	23	Deep wound	*R* > 2 16 (16)	*R* > 2 32 (64)	*R* > 8 256 (256)	*R* > 8 Growth at 64	*R* > 0.5 Growth at 16
*E. coli*	114	Deep wound	S ≤ 2 0.5 (0.5)	*R* > 2 16 (16)	*R* > 8 Growth at 512	*R* > 8 Growth at 64	*R* > 0.5 Growth at 16

Green solid = sensitive, Blue = no BP but low minimum inhibitory concentration, Yellow = susceptible, Orange = no BP but high minimum inhibitory concentration, Red= resistant.

* Enterococci are resistant to aminoglycosides when used in monotherapy.

*A. baumannii*, *Acinetobacter baumannii*; BP, breakpoint; E. coli, *Escherichia coli*; I, increased exposure; *K. pneumoniae*, *Klebsiella pneumoniae*; MRSA, methicillin-resistant *Staphylococcus aureus*; *P. aeruginosa*, *Pseudomonas aeruginosa*; R, resistant; S, sensitive.

### Antimicrobial resistance of included strains

The MIC and minimum bactericidal concentration (MBC) of antimicrobials used in combat trauma prophylaxis and the treatment of musculoskeletal infection were determined using the well-established broth microdilution methods.^49^ In summary, a 96-well microtitre plate was used to prepare a range of antimicrobial concentrations in 100 µl of Mueller-Hinton Broth 2 (MHB) via a series of two-fold serial dilutions. Bacteria were diluted to 10^6^ colony-forming units (CFU) ml^-1^ in MHB, and 100 µl was used to seed each well of the microtitre plate to give a final inoculation density of 5 × 10^5^ CFU ml^-1^. Plates were incubated statically at 37°C for 18 hours in air. The MIC was defined as the lowest antimicrobial concentration at which there was no visible growth of bacteria. The extent of bacterial growth after incubation was also determined by obtaining OD_595nm_ measurements using a Bio-Rad iMark microplate absorbance reader (Bio-Rad Laboratories, USA). MBC was determined by culturing 10 µl of the MIC well, along with adjacent more concentrated conditions for 24 hours on Mueller-Hinton agar. The MBC was the lowest concentration condition with > 3-log_10_ reduction in CFU counts. Breakpoints were defined as per the EUCAST clinical breakpoints tables (v. 16.0).^45^

### Preparation of PerOssal beads

PerOssal beads were prepared by soaking in antimicrobial solutions for two minutes following manufacturer instructions (Table II, Figure 1), resulting in all fluid being absorbed.^50^ For antimicrobials not listed in the manufacturer’s guidance, the standard intravenous dose was dissolved in 10 ml phosphate-buffered saline (PBS).

**Table II.** Description of antimicrobials loaded into PerOssal beads (OSARTIS, Germany).

Antimicrobial(s)	Concentration	Rationale	Manufacturer
Tobramycin	40 mg ml^-1^	Manufacturer instructions	Merck, Germany
Gentamicin	40 mg ml^-1^	Manufacturer instructions	Gibco, Thermo Fisher Scientific, USA
Cefazolin	100 mg ml^-1^	Ukrainian combat trauma prophylaxis guideline (intravenous) The powdered intravenous dose dissolved in 10 ml PBS	Sigma-Aldrich, USA
Amoxicillin + Clavulanic acid (Co-amoxiclav)	120 mg ml^-1^	UK combat trauma prophylaxis guideline (intravenous) The powdered intravenous dose dissolved in 10 ml	Sigma-Aldrich, USA
Gentamicin + Rifampicin	50 / 60 mg ml^-1^	Manufacturer instructions	Gibco (USA)/Bioserv (UK)

PBS, phosphate-buffered saline.

### Zone of inhibition testing

The effect of antimicrobial-laden PerOssal beads was assessed against isolates using a modified zone of inhibition assay adapted from the EUCAST disc diffusion guidance to permit testing of this local antimicrobial carrier.^51^ The bacterial inoculum was prepared as standard by diluting stationary-phase cultures 1:100 in MHB and spreading 250 µl onto MHB agar using a L-shaped spreader. Antimicrobial-laden beads were placed into agar wells punched to match the bead diameter. One bead was placed per agar plate. Negative controls confirmed biomaterial and reagent sterility. No-treatment control assessed unimpeded bacterial growth. Plates were incubated at 37°C for 18 hours in air. Zones of inhibition were measured with a ruler, taking the mean of four perpendicular points. Plates were then reincubated for seven days to assess for regrowth into the zone of inhibition which may indicate a transient effect or spontaneous resistance.^52,53^

### Time-kill analyses

Time-kill analysis followed methods adapted from the CLSI M26 guidance.^54^ Bacteria were added to MHB at a final concentration of 10⁵ CFU ml⁻¹. This was combined with one antimicrobial-laden PerOssal bead in 150 µl MHB. Negative controls confirmed biomaterial and reagent sterility. No-treatment control assessed unimpeded bacterial growth. Cultures were incubated at 37°C with shaking at 180 RPM. At four, 24, and 48 hours, aliquots were taken, serially diluted in PBS, and plated on MHB agar for CFU enumeration. The primary outcome was bacterial growth at 48 hours.

### Statistical analysis

Experiments were performed on at least three independent occasions, and the resulting data were presented as the arithmetic mean (SD) of these biological repeats. Conditions within each time-kill experiment were compared using a two-way analysis of variance (ANOVA) with repeated measures and Bonferroni post hoc analysis for multiple comparisons. The significance threshold was set at p < 0.05 and is reported in figures for the primary outcome, i.e. bacterial growth at 48 hours. Statistical analysis was performed on Prism 10 (GraphPad Software, USA).

## Results

### Clinical isolates from Ukraine are multidrug-resistant

Gram-negative bacterial isolates from the Russo-Ukraine conflict were tested for their susceptibility to antimicrobials commonly used in combat trauma prophylaxis and the treatment of musculoskeletal infections. All isolates exhibited resistance to the test antimicrobials gentamicin, tobramycin, co-amoxiclav, cefazolin, and ciprofloxacin with MICs far exceeding breakpoints reported in the EUCAST clinical breakpoint tables (Table I, Supplementary Table 1).^49^ In more than ten cases, bacterial growth persisted at 512 mg L^-1^ of antimicrobial (Table I).

### Beads loaded with combination treatments are more potent than single agents

**Fig. 2:**
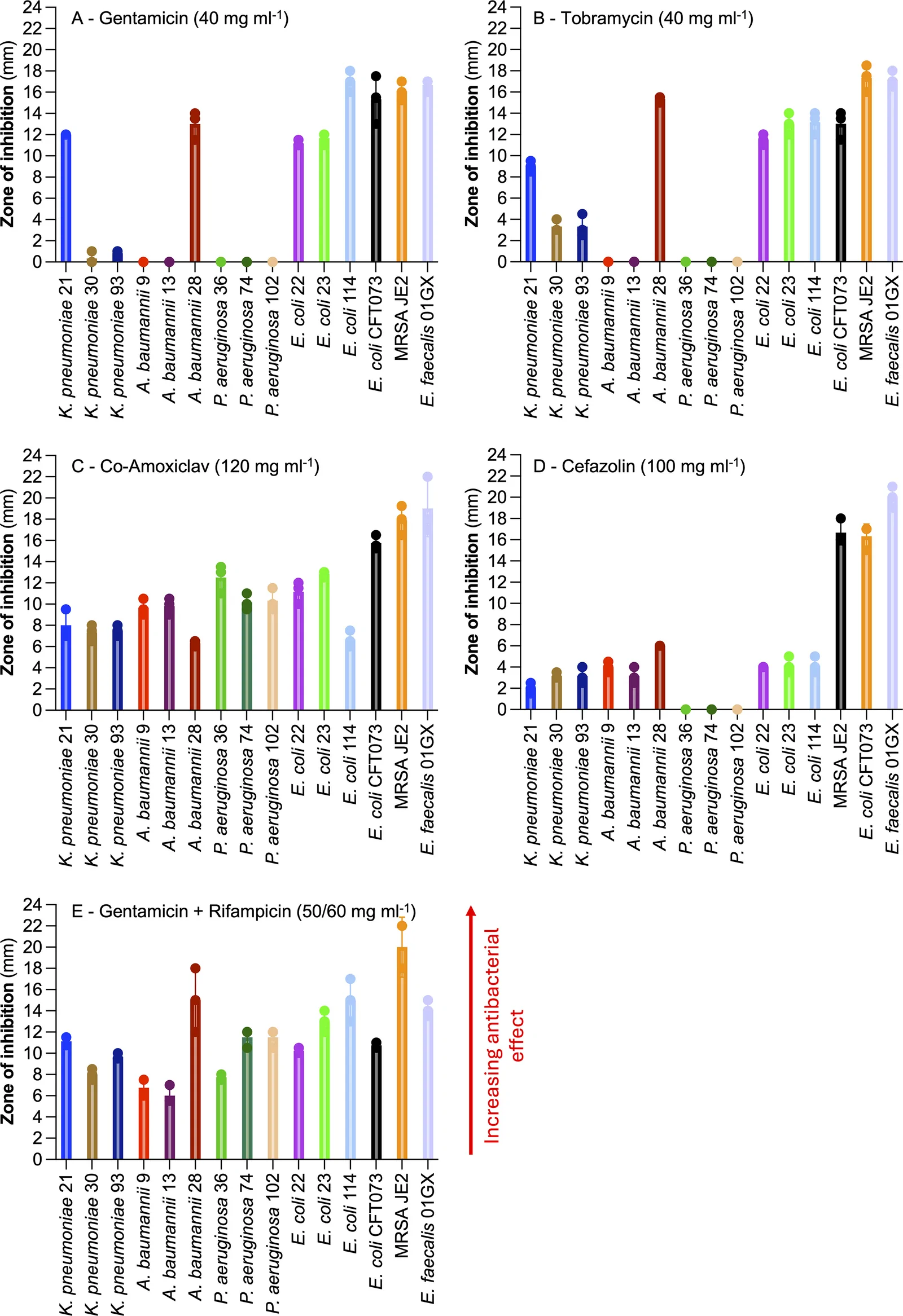
a) to e) Zones of inhibition for antimicrobial soaked PerOssal beads (OSARTIS, Germany) against extensively drug-resistant (XDR) Gram-negative bacteria associated with conflict wounds in Ukraine or additional clinical strains. All experiments were replicated in three independent assays. Error bars show the SD of the mean. MRSA, methicillin-resistant *Staphylococcus aureus*.

The growth inhibitory activity of antimicrobial-laden PerOssal beads was assessed using the zone of inhibition assay. Zones of inhibition were small or absent across XDR conflict-associated bacterial strains for beads loaded with gentamicin, tobramycin, or cefazolin (Figure 2). During the pilot phase, vancomycin was assessed against each Gram-negative species, with data confirming a lack of clinically significant antimicrobial activity against drug-resistant Gram-negative bacteria consistent with its inability to cross the outer membrane (Supplementary Figure 1). Co-amoxiclav showed broad antimicrobial effects against all strains. Gentamicin and rifampicin in combination showed broad antimicrobial effects against all strains, greater than that seen with gentamicin alone (Figure 2, panel E). All antimicrobials tested showed increased activity against additional clinical strains. Overall, zone diameters mirrored MIC-based EUCAST interpretations. Re-growth > 1 mm into the zone of inhibition was not seen at day seven for any condition.

### Combination drug loaded beads show bactericidal activity against XDR conflict isolates

**Fig. 3:**
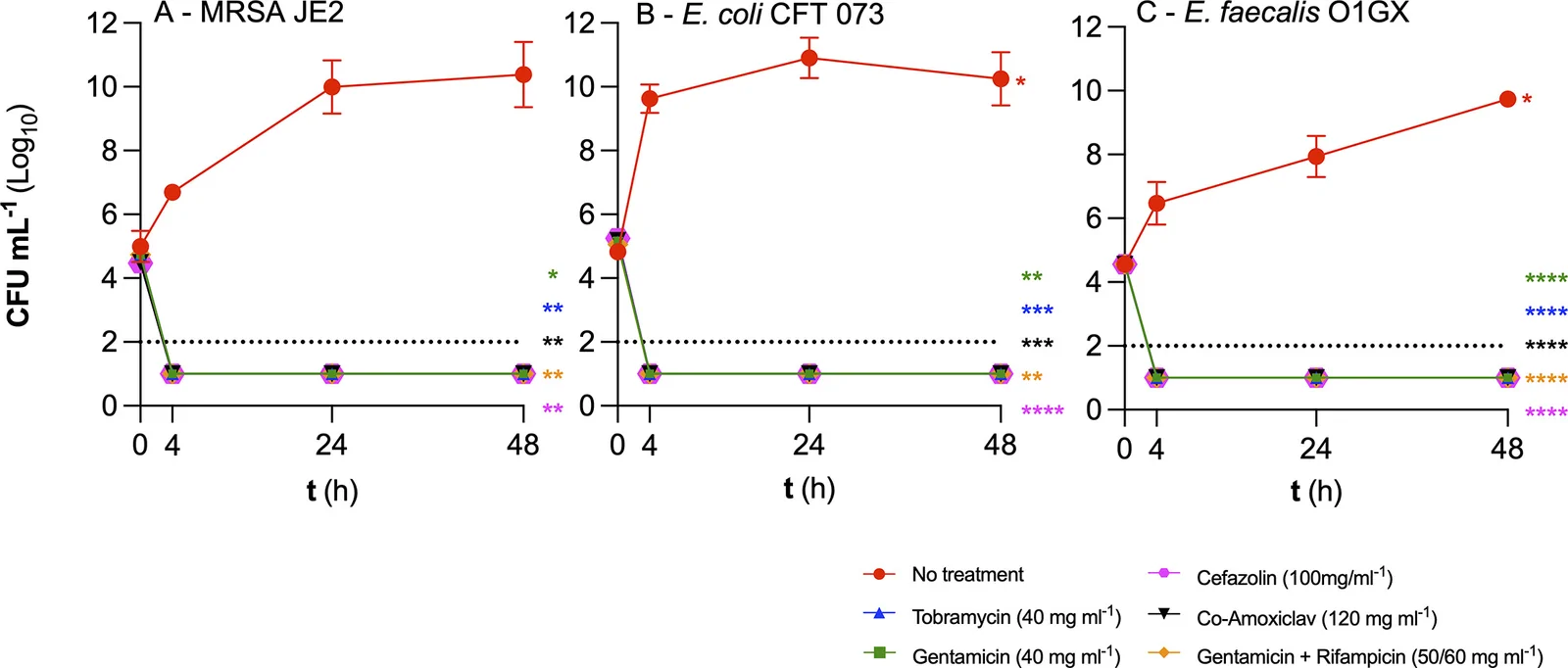
a) to c) Time-kill analyses for antimicrobial soaked PerOssal beads (OSARTIS, Germany) against additional clinical strains. Three independent repeats. All experiments were replicated in three independent assays. Error bars show the SD of the mean. Timepoints were compared points using a two-way analysis of variance with Bonferroni correction for multiple comparisons. Significance reported for 48-hour timepoint relative to inoculum. *p ≤ 0.05, **p ≤ 0.01, ***p ≤ 0.001, ****p ≤ 0001. Dotted line = limit of detection. CFU, colony-forming units; E. coli, *Escherichia coli*; MRSA, methicillin-resistant *Staphylococcus aureus*.

**Fig. 4:**
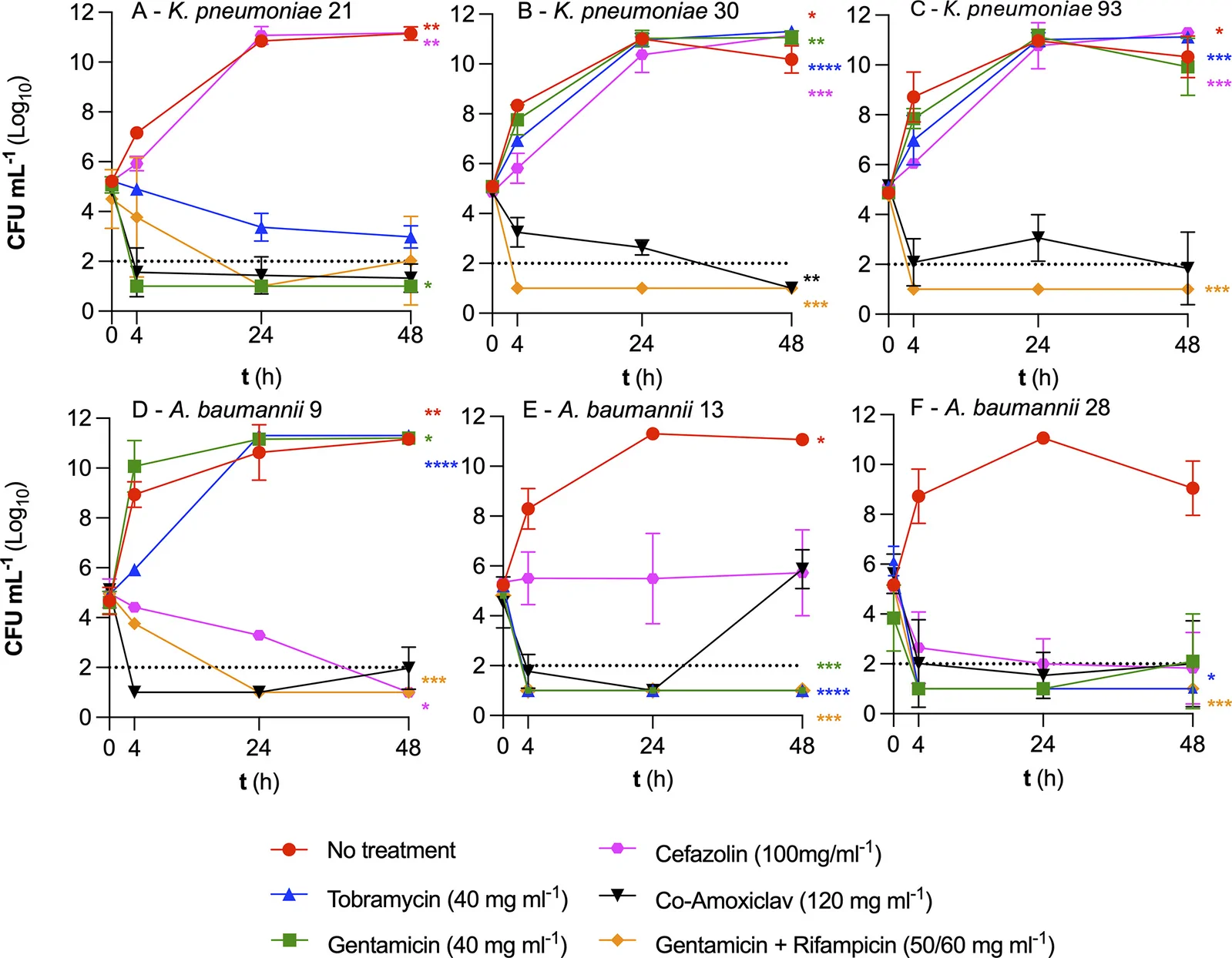
a) to f) Time-kill analyses for antimicrobial soaked PerOssal beads (OSARTIS, Germany) against extensively drug-resistant (XDR) Gram-negative bacteria (*Klebsiella pneumoniae* (*K. pneumoniae)* and *Acinetobacter baumannii* (*A. baumannii*)) associated with conflict wounds in Ukraine. All experiments were replicated in three independent assays. Error bars show the SD of the mean. Timepoints were compared points using a two-way analysis of variance with Bonferroni correction for multiple comparisons. Significance reported for 48-hour timepoint relative to inoculum. *p ≤ 0.05, **p ≤ 0.01, ***p ≤ 0.001, ****p ≤ 0001. Dotted line = limit of detection. CFU, colony-forming units.

**Fig. 5:**
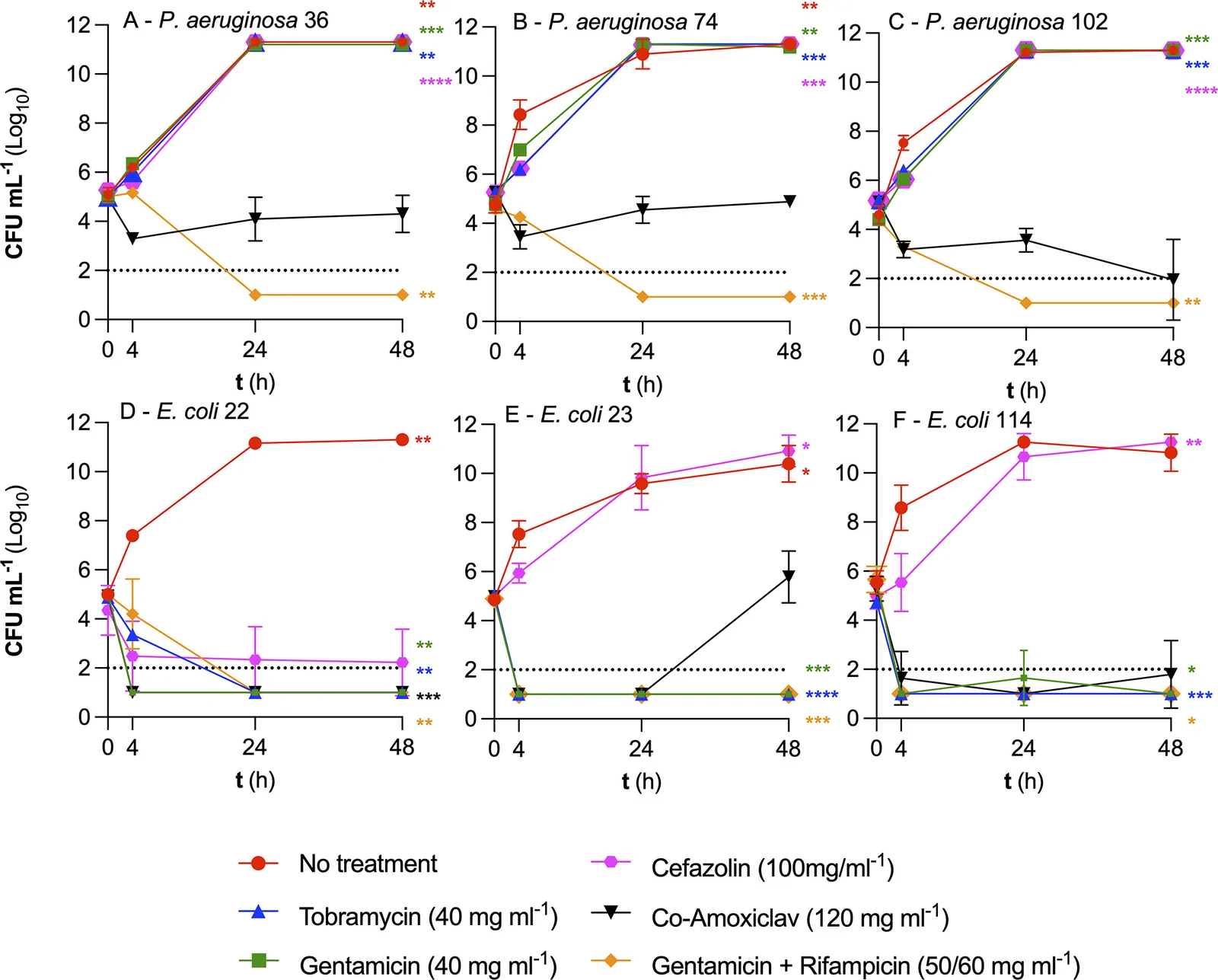
a) to f) Time-kill analyses for antimicrobial soaked PerOssal beads (OSARTIS, Germany) against extensively drug-resistant (XDR) Gram-negative bacteria (*Pseudomonas aeruginosa* (*P. aeruginosa*) and *Escherichia coli* (*E. coli*)) associated with conflict wounds in Ukraine. All experiments were replicated in three independent assays. Error bars show the SD of the mean. Timepoints were compared points using a two-way analysis of variance with Bonferroni correction for multiple comparisons. Significance reported for 48-hour timepoint relative to inoculum. *p ≤ 0.05, **p ≤ 0.01, ***p ≤ 0.001, ****p ≤ 0001. Dotted line = limit of detection. CFU, colony-forming unit.

Time-kill analyses assessed the bactericidal effects of antimicrobial-laden PerOssal beads. Against additional clinical strains, all antimicrobials showed increased activity (Figure 3). Against XDR isolates, clear differences between regimens were observed (Figure 4 and Figure 5). Combination therapy with gentamicin and rifampicin produced the most consistent effect, achieving bacterial killing across all species tested. Co-amoxiclav inhibited growth in all XDR isolates, with bactericidal activity observed in selected *K. pneumoniae* and *E. coli* strains. Aminoglycoside monotherapy (gentamicin or tobramycin) demonstrated consistent killing of *E. coli* but variable activity against *K. pneumoniae*, *A. baumannii*, and *P. aeruginosa*. Cefazolin showed no activity against *K. pneumoniae* or *P. aeruginosa*, with inconsistent effects in *A. baumannii* and *E. coli*.

## Discussion

This study demonstrates the potential therapeutic value of antimicrobial-laden PerOssal beads against bacteria associated with heavily contaminated open fractures, including XDR organisms from the Russo-Ukraine war. These findings suggest that antimicrobial effectiveness in these injuries depends on achieving sufficient local exposure rather than antimicrobial class alone. The greatest activity was observed with gentamicin and rifampicin in combination, where broad-spectrum bacterial killing was observed. We hypothesize two potential mechanisms are important: 1) aminoglycosides may cause membrane disruption that promotes rifampicin entry into the bacterial cell; and 2) the combination of inhibition of transcription (rifampicin) and protein synthesis (gentamicin) may inhibit the ability of the bacterium to produce proteins more than either agent alone.^55^ Co-amoxiclav inhibited the growth of all XDR species, with killing observed against certain strains of XDR *K. pneumoniae* and XDR *E. coli*. While there is no compelling clinical evidence that bactericidal activity is superior to bacteriostatic activity, our data indicate that gentamicin and rifampicin in combination may provide the most potent approach to prophylactic treatment of conflict wounds.^56^ Notably, high-dose co-amoxiclav was bacteriostatic against *P. aeruginosa* and *A. baumannii*, despite not being routinely used against these pathogens. Its inclusion is justified by UK military guidelines, which recommend it for trauma prophylaxis.^20,45^

Directly administering powdered antimicrobial drug leads to approximately two hours of local effect, before washout.^57^ In contrast, the use of carriers allows for sustained release above bacterial MICs for several days.^42^ The PerOssal system is established as a treatment for bone infection, and is in routine use in the UK and Europe.^43,44^ The potential benefits of local antimicrobial delivery must be balanced against potential risks of local cytotoxicity, systemic toxicity, and incomplete killing of bacteria, driving further resistance. However, no convincing evidence of these adverse events has been identified.^34-40^

We did not include vancomycin; while this is an effective treatment for Gram-positive infections, it has no activity against Gram negatives because the outer membrane of these organisms blocks access to the antimicrobial’s target. Vancomycin would, therefore, not provide the basis for a suitable prophylactic approach for conflict wounds.^33^ This study showed promising results for gentamicin and rifampicin in combination, and this antimicrobial drug combination is recommended in the PerOssal manufacturer guidelines.^50^ Rifampicin monotherapy is generally avoided in bacterial infection due to rapid resistance selection through single-step mutations, occurring in both Gram-positive and Gram-negative organisms.^58^ For this reason, rifampicin monotherapy was not assessed.

Infection risk following open fracture is strongly influenced by the interval between injury and surgical debridement. In conflict settings, this delay may occur because evacuation routes are denied and casualties are held at forward medical facilities for prolonged periods.^4^ When combined with high rates of MDR/XDR bacterial contamination, these delays have contributed to the severe infection burden seen in the Russo-Ukraine war.^10^ This may be a causative factor in the high incidence of amputation in this conflict.^59-61^ A similar situation occurs in civilian disasters such as earthquakes, where crush injuries, environmental contamination, and overwhelmed healthcare systems prolong the time to operative wound control.^6,7^ During this period, systemic antimicrobial may be administered, but devascularized tissue and high bacterial burden limit effective antimicrobial exposure at the wound surface. Local antimicrobials applied during initial care may therefore reduce infection risk in both military and humanitarian contexts, and reduce morbidity in difficult-to-treat limb infections. This approach may also benefit civilian patients in low- and middle-income countries, where high rates of open fracture related infection and amputation have been observed.^62,63^

Further research involving contaminated wound and open fracture models is needed to define how local antimicrobial delivery may prevent MDR/XDR infection after conflict injury. Studies in models of established infection are also required to determine whether local delivery can treat MDR/XDR musculoskeletal infection with biofilm. Future studies may be in vitro with flow cell models, ex vivo, or in vivo. Local antimicrobial delivery is well established in orthopaedic surgery, and several licensed systems are available, so introducing these approaches to military medical teams may be justified. This may be as part of an observational or interventional study, although the challenges of conducting studies during conflict are recognized. Such studies may be particularly relevant to disaster response and austere trauma systems, where definitive surgery cannot be delivered promptly. Biomaterials research should optimize antimicrobial carriers to provide additional innate antimicrobial activity. Porous bioactive glass beads or wool-like preparations loaded with antimicrobial drug may outperform inert carriers such as calcium sulphate, particularly in the context of AMR. S53P4 bioactive glass has shown promise for osteomyelitis and infected nonunion, when applied alongside systemic antimicrobials.^64^

The collaboration between Ukraine and the UK is a strength of this study because it enabled direct investigation of conflict-associated bacteria that cause significant morbidity and mortality. Civilian–military partnership, supported by interdisciplinary work between microbiologists, clinicians, and material scientists, brings complementary perspectives to the problem of conflict injury. This study has limitations inherent to in vitro work, such as artificial aggregate growth, differences in antibiotic diffusion rates in agar, and the lack of complexity found in conflict wounds. Furthermore, vascular circulation may change antimicrobial concentration over time in vivo. Bacterial species were studied individually, whereas conflict wound infections may be polymicrobial. This study establishes proof-of-concept activity at manufacturer-recommended or standard intravenous loadings. Drug elution from the PerOssal beads and subsequent pharmacodynamics were not assessed. The XDR isolates were collected from the Russo-Ukraine war in 2024. Although resistance patterns may vary over time and between regions, these findings are likely to remain relevant to conflict-associated wounds.^9,14^ Despite these limitations, in vitro work forms an important first step before clinical study and widespread use.

In summary, antimicrobial-laden PerOssal hydroxyapatite and calcium sulphate beads display in vitro killing of XDR Gram-negative bacteria associated with conflict wounds in Ukraine. Local antimicrobial delivery close to the point of injury may represent an important intervention to reduce morbidity and mortality associated with infection after heavily contaminated open fractures, particularly when surgical debridement is delayed. The first dose could be applied immediately after injury as an adjunct to systemic prophylaxis, with further application during prolonged field care, and between re-look operations prior to definitive soft-tissue coverage. This is relevant to both modern conflict and civilian disaster settings, where timely operative care cannot be guaranteed.

## Data Availability

All data generated or analyzed during this study are included in the published article and/or in the supplementary material.
